# Tuberculosis and social aspects in a Brazilian state of high vulnerability: a systematic review

**DOI:** 10.1590/0034-7167-2025-0391

**Published:** 2026-08-21

**Authors:** Elivelton Sousa Montelo, Amanda Silva de Oliveira, Zulimar Márita Ribeiro Rodrigues, Ana Hélia de Lima Sardinha

**Affiliations:** I Universidade Federal do Maranhão. São Luís, Maranhão, Brazil

**Keywords:** Tuberculosis, Health Status Disparities, Social Determinants of Health, Health Services Accessibility, Systematic Reviews as Topic., Tuberculosis, Inequidad en Salud, Determinantes Sociales de la Salud, Acceso a los Servicios de Salud, Revisión Sistemática.

## Abstract

**Objectives:**

to synthesize evidence on social, economic, territorial, and healthcare access factors associated with the occurrence and outcomes of tuberculosis in Maranhão.

**Methods:**

a mixed-methods systematic review, registered in PROSPERO and conducted in accordance with PRISMA 2020. The search was carried out in May 2025 using the SPIDER strategy. Original studies published between 2014 and 2025, in Portuguese, English, or Spanish, were included; duplicates and studies not focused on the target population were excluded. Methodological quality and risk of bias were assessed using the Mixed

**Methods:**

Appraisal Tool.

**Results:**

of the 94 studies identified, 12 were included. Four thematic axes were identified: spatial distribution and risk areas; sociodemographic vulnerabilities; organization and access to health services; and treatment conditions and social protection. Structural inequalities were associated with higher incidence, treatment abandonment, and barriers to diagnosis.

**Conclusions:**

the persistence of tuberculosis in Maranhão is related to social and territorial vulnerabilities, requiring intersectoral policies, strengthening of primary healthcare, and monitoring of social indicators.

## RESUMEN

**Objetivos:**

sintetizar evidencias sobre factores sociales, económicos, territoriales y de acceso a los servicios de salud asociados con la ocurrencia y los desenlaces de la tuberculosis en Maranhão.

**Métodos:**

revisión sistemática de métodos mixtos, registrada en PROSPERO y realizada de acuerdo con las directrices PRISMA 2020. La búsqueda se llevó a cabo en mayo de 2025, utilizando la estrategia SPIDER. Se incluyeron estudios originales publicados entre 2014 y 2025, en portugués, inglés o español; se excluyeron duplicados y estudios que no se centraban en la población objetivo. La calidad metodológica y el riesgo de sesgo se evaluaron mediante el Mixed

**Resultados:**

de los 94 estudios identificados, se incluyeron 12. Se identificaron cuatro ejes temáticos: distribución espacial y áreas de riesgo; vulnerabilidades sociodemográficas; organización y acceso a los servicios de salud; y condiciones y protección social en el tratamiento. Las desigualdades estructurales se asociaron con una mayor incidencia, abandono del tratamiento y barreras en el diagnóstico.

**Conclusiones:**

la persistencia de la tuberculosis en Maranhão se relaciona con vulnerabilidades sociales y territoriales, lo que exige políticas intersectoriales, el fortalecimiento de la atención primaria y el monitoreo de indicadores sociales.

## INTRODUCTION

Tuberculosis (TB) is an infectious disease caused by Mycobacterium tuberculosis that primarily affects the lungs, although it may also involve other organs and systems. Transmission occurs via the airborne route through exposure to aerosols expelled by individuals with active pulmonary TB^(1)^. In 2022, TB remained among the leading causes of death from infectious diseases worldwide, with 10.6 million new cases and 1.3 million deaths, according to the World Health Organization^(2)^.

In Brazil, TB continues to have a significant impact on public health. In 2023, more than 80,000 new cases were reported, resulting in an incidence rate of 37.0 per 100,000 inhabitants, in addition to 5,845 deaths^(3)^. Despite the expansion of diagnosis and treatment, the disease remains concentrated in territories marked by social and structural inequalities, such as poverty, inadequate housing, and barriers to accessing healthcare services^(4)^. In the context of Maranhão, these conditions are even more pronounced. The state presents high levels of poverty, low educational attainment, precarious housing conditions, and limited access to sanitation and primary healthcare, factors that favor transmission and hinder TB control^(5)^. In 2023, 2,808 cases were reported, leading the Ministry of Health to include the state among those prioritized for TB control actions^(6)^.

Although the literature points to associations between social determinants and TB, the available studies in Maranhão are scattered, heterogeneous, and generally focused on isolated aspects^(7,8)^, sometimes epidemiological, exclusively socioeconomic, or related to the organization of services. No integrated syntheses were identified that allow for a comprehensive understanding of how social, economic, territorial, and healthcare access factors simultaneously influence TB outcomes in this state. These scientific gaps ultimately limit the ability of managers and professionals to formulate public policies based on local evidence that are capable of addressing the contexts of vulnerability surrounding TB.

## OBJECTIVES

To synthesize available evidence on social, economic, territorial, and healthcare access factors associated with the occurrence and outcomes of TB in populations in Maranhão.

## METHODS

### Study design and protocol

This is a mixed-methods systematic review, registered in the International Prospective Register of Systematic Reviews (PROSPERO)^(9)^ under the number CRD42020214295, and conducted in accordance with the Preferred Reporting Items for Systematic Reviews and Meta-Analyses (PRISMA) 2020 guidelines^(10)^.

### SPIDER strategy and guiding question

The research question was structured based on the SPIDER strategy^(11)^, which is suitable for reviews including qualitative and mixed-methods data:

- S (Sample): Populations affected by tuberculosis in the state of Maranhão;
- PI (Phenomenon of Interest): Social, economic, territorial, epidemiological, and healthcare access factors;
- D (Design): Qualitative, quantitative, and mixed-methods studies;
- E (Evaluation): Perceptions, experiences, associations, or effects attributed to the factors studied;
- R (Research type): Publications in Portuguese, English, or Spanish.

The search was conducted in May 2025 in the PubMed/MEDLINE, Scopus, SciELO, PsycINFO, Embase, and Virtual Health Library (BVS in Portugal) databases (Chart 1). According to the SPIDER strategy, the following guiding question was formulated: Which social, economic, territorial, and healthcare access factors are associated with the occurrence and outcomes of tuberculosis, such as incidence, mortality, treatment adherence, treatment abandonment, and timely diagnosis, in Maranhão, a Brazilian state with high endemicity, according to evidence published between 2014 and 2025?

**Chart 1:** Search strategies used in the databases, São Luís, Maranhão, Brazil, 2025

Database	Applied search strategy	Field/filter applied
PubMed/MEDLINE	Tuberculosis[Title/Abstract] AND Maranhão[Title/Abstract]	Title/Abstract; no study filter
Scopus	TITLE-ABS(Tuberculosis^*^) AND TITLE-ABS(Maranhão)	Title/Abstract; no filter
SciELO	Tuberculose AND Maranhão	Standard search field; language: Portuguese/Spanish/English
PsycINFO	TI(Tuberculosis^*^) AND TI(Maranhão)	Title/Abstract; no filter
Embase	‘tuberculosis’:ab,ti AND ‘Maranhao’:ab,ti	Title/Abstract; filter: humans
BVS (Virtual Health Library)	tw:(Tuberculose^*^) AND tw:(Maranhão)	Title/Abstract (tw); filter: humans; 2014-2025

Note: When applicable, truncation (^*^) was used to expand the retrieval of related terms, according to the syntax of each database.

Broader preliminary strategies were tested; however, the final search prioritized terms applied to the title and abstract, such as Tuberculosis AND Maranhão, due to greater sensitivity. The search logic was adapted to the other databases according to the syntax of each. The search was conducted using the SPIDER strategy, with controlled and uncontrolled descriptors combined using Boolean operators (AND, OR) and truncation (*). The terms were applied to the title and abstract, an option adopted to increase specificity and avoid excessive retrieval of irrelevant studies, given the broad conceptual dispersion related to the social determinants of health.

### Inclusion and exclusion criteria

Primary studies on TB were included, with qualitative, quantitative, or mixed-methods designs, conducted exclusively in the state of Maranhão, published between January 2014 and May 2025, in Portuguese, English, or Spanish, that addressed social, economic, territorial, or healthcare access factors related to the disease in human populations. The time frame from 2014 to 2025 was adopted to encompass the most recent decade of publications and to allow the analysis of recent evidence, including studies produced after the most recent updates to the National Tuberculosis Control Plan (PNCT)^(3)^.

Duplicate studies, reviews, theoretical essays, editorials, and unpublished theses and dissertations were excluded, as were studies with a strictly clinical, laboratory, or biomolecular focus without an interface with social or contextual factors; studies aimed exclusively at the development or validation of instruments; studies involving highly specific populations, such as individuals with rare comorbidities, whose analysis would hinder the interpretation of social determinants in a broader sense; and articles that did not meet minimum methodological quality criteria, as assessed using the Mixed Methods Appraisal Tool (MMAT)(2018 version).

### Study selection

Study selection was conducted in three successive stages: reading of titles, abstracts, and full texts. The process was carried out independently by two reviewers (E.S.M. and A.H.L.S.), with disagreements resolved by a third researcher (Z.M.R.R.). Duplicate records were previously identified and removed using Mendeley^©^ software, ensuring greater accuracy in the screening process. The processes of identification, screening, eligibility, and final inclusion are detailed in the flowchart developed in accordance with the PRISMA 2020 guidelines.

### Methodological quality assessment

The methodological quality and risk of bias of the studies were assessed using the MMAT, 2018 version^(12)^. This tool applies specific criteria according to study design, considering aspects such as clarity of objectives, methodological adequacy, sample representativeness, validity of measures, and consistency between results and conclusions.

The researchers involved underwent prior training using a standardized Microsoft Excel^®^ spreadsheet to ensure uniform application of the criteria. The assessment was conducted independently by two reviewers, followed by discussion to reach consensus in cases of disagreement. The choice of the MMAT is justified by its scope and applicability in systematic reviews that integrate different methodological approaches.

### Data extraction and analysis

Data were extracted into a standardized Microsoft Excel^®^ spreadsheet containing authors, year of publication, study location, study design, methodological approach, population, variables investigated, main findings, and MMAT assessment. Extraction was performed in a blinded manner by two reviewers who received prior training in the use of the spreadsheet, ensuring uniformity of the process and resolution of discrepancies by consensus, with the participation of a third researcher when necessary.

The analysis followed the method proposed by Braun and Clarke^(13,14)^, structured into six stages: (1) familiarization with the results of the included studies; (2) generation of initial codes; (3) searching for themes; (4) reviewing themes; (5) defining and naming themes; and (6) synthesis of findings in a report. For quantitative studies, a descriptive synthesis of the main indicators (incidence, mortality, adherence, abandonment, and timely diagnosis) was performed and presented in tables. Qualitative and mixed-methods studies were integrated through interpretative analysis. Due to the heterogeneity of study designs and outcomes, a meta-analysis was not feasible.

## RESULTS

**Figure 1:**
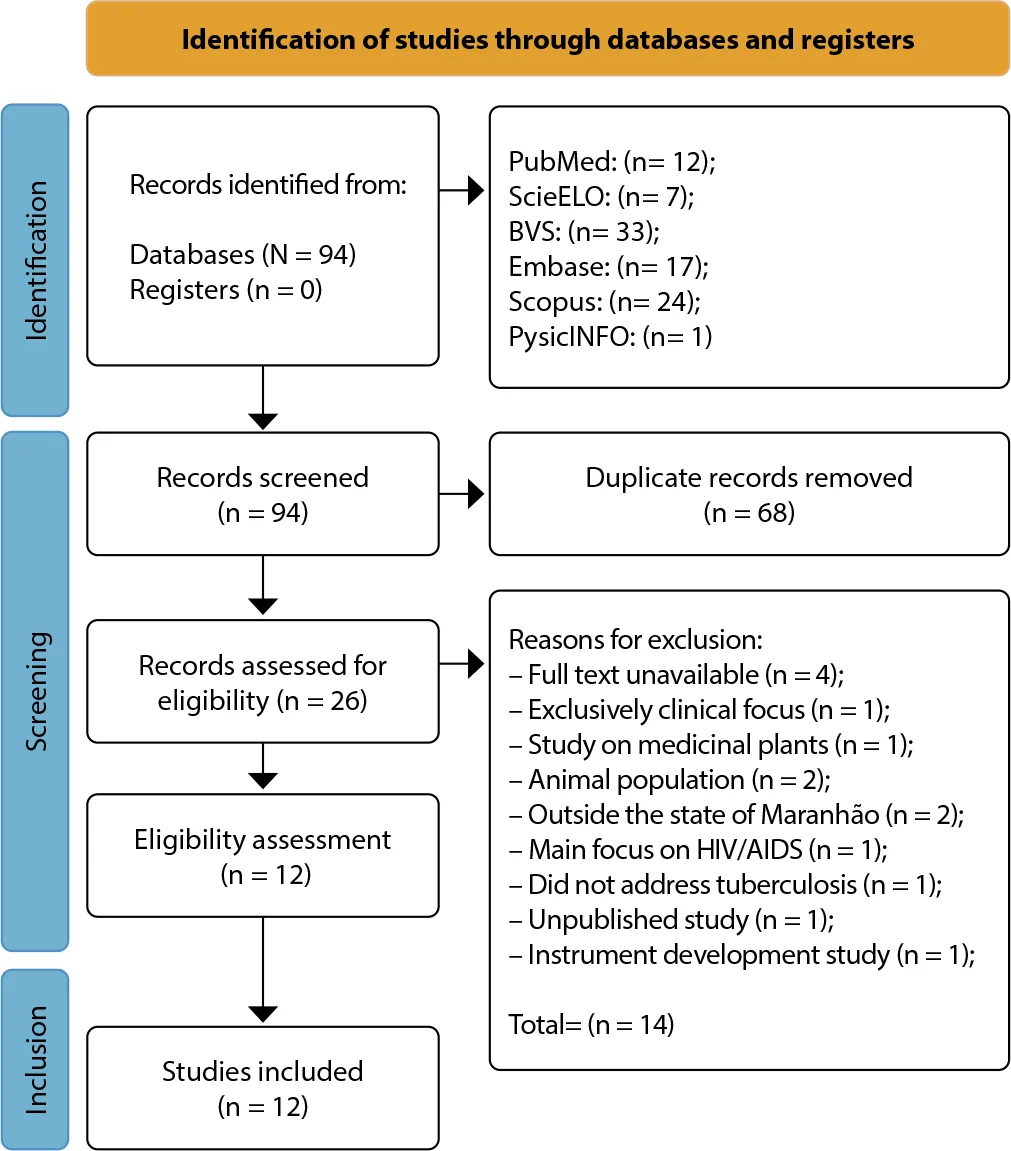
Flowchart of study selection, developed based on the Preferred Reporting Items for Systematic Reviews and Meta-Analyses recommendations, São Luís, Maranhão, Brazil, 2025

A total of 94 studies were identified in the selected databases. After the removal of 68 duplicates, 26 articles were screened through title and abstract reading. Of these, 14 were excluded for not meeting the eligibility criteria, resulting in 12 studies included in the review. The selection process is detailed in the PRISMA flowchart (Figure 1).

Of the 12 studies included in the present review, 11 had a quantitative design, including five ecological studies, four of which used spatial analysis, four cross-sectional studies, and one case-control study. In addition, one study with a mixed-methods approach was identified. Although searches were conducted starting from 2014, the eligible studies included in the final sample were published between 2015 and 2025. Regarding the language of publication, two articles were available only in Portuguese, six in English, and four in both languages, with none published in Spanish. Methodological quality was assessed based on MMAT scores, with six studies achieving 100%, five achieving 80%, and one achieving 60%. The main methodological and thematic characteristics of the included studies are systematized in Chart 2, including information on authorship, study setting, design and sample size, variables investigated, main results, and methodological quality assessed using the MMAT.

**Chart 2:** Methodological characteristics and main results of the studies included in the review

Authorship (year)	Territory	Design / number of participants	Main variables	Main results	Quality (MMAT)
Santos Neto et al. (2015)^(15)^	São Luís-MA	Ecological with spatial analysis; n=221 deaths (2008-2012)	Spatial and space-time clusters of TB deaths	Significant clusters identified; RR up to 3.87	80%
Silva et al. (2015)^(16)^	Priority municipalities of Maranhão	Cross-sectional; n=2,277 cases (2005-2010)	Factors associated with re-entry and abandonment	Men aged 20-39, <8 years of schooling, alcohol use associated	100%
Pereira et al. (2018)^(17)^	Maranhão	Cross-sectional; n=4,553 cases (2001-2011)	Factors associated with TB/HIV coinfection	Coinfection prevalence 15.1%; male sex and low schooling associated	100%
Costa et al. (2018)^(18)^	Maranhão	Ecological; n=1,706 new TB cases and 1,365 PHC units (2012)	PHC structure and TB detection	Better PHC structure associated with higher detection (IDR=1.61)	100%
Silva et al. (2019)^(19)^	Maranhão	Cross-sectional; n=1,505 older adults (2010-2015)	Sociodemographic and clinical profile	Low schooling, diabetes, lack of HIV testing associated	100%
Andrade et al. (2020)^(20)^	Imperatriz-MA	Ecological with spatial analysis; n=413 cases (2013-2018)	Spatial distribution and outcomes	Higher density of outcomes in central areas	80%
Müller et al. (2021)^(21)^	Caxias-MA	Cross-sectional; n=100 patients (2018)	Access to TB treatment	High access to care and medications	60%
Sousa et al. (2021)^(22)^	Imperatriz-MA	Ecological with spatial analysis; n=721 cases (2009-2018)	Territorial vulnerability to TB-DM	High-risk clusters identified	80%
Aragão et al. (2023)^(23)^	São Luís-MA	Cross-sectional; n=7,948 cases (2010-2020)	Health system organization for TB diagnosis	Hospital-based diagnosis predominated	80%
Santos et al. (2023)^(24)^	Maranhão	Ecological with spatial analysis; n=949 deaths (2010-2015)	Socioeconomic indicators and TB deaths	Deaths concentrated in North and central regions	80%
Aragão et al. (2024)^(25)^	São Luís-MA	Sequential explanatory mixed-methods; n=7,958 cases (2010-2019)	Risk areas and social protection	Social benefits associated with improvement	100%
Mendes et al. (2025)^(26)^	São Luís-MA	Case-control; n=138 individuals	Nutritional factors and food insecurity	TB associated with low weight and food insecurity	100%

*APS - Primary Health Care; DM - Diabetes mellitus; HIV - Human Immunodeficiency Virus; CI - Confidence interval; IDR - Incidence density ratio; BMI - Body Mass Index; MA - Maranhão; OR - Odds ratios; RR - Relative risk; TB - Tuberculosis; TB-DM - Tuberculosis and diabetes mellitus; TB/HIV - Tuberculosis and Human Immunodeficiency Virus; UBSs - Primary Health Care Units.*

Based on the thematic analysis by Braun and Clarke^(13,14)^ and considering the Phenomenon of Interest (PI) component of the SPIDER strategy^(11)^, centered on social, economic, territorial, epidemiological, and healthcare access factors, the results were organized into four axes. The process involved coding, grouping, and cyclical review of the data, allowing the identification of recurring patterns and the synthesis of determinants influencing the occurrence, diagnosis, and treatment of TB in Maranhão. The identified axes were: (1) Spatial distribution and risk areas; (2) Sociodemographic vulnerabilities; (3) Access to and organization of health services; and (4) Conditions and social protection in treatment.

### Axis 1 - Spatial distribution and risk areas

Spatial studies showed an uneven concentration of TB cases and deaths. In São Luís, mortality clusters were observed in specific periods and regions^(15)^. In the city of Imperatriz, there was a higher density of cure, treatment abandonment, and death in vulnerable central areas, as well as risk clusters for TB associated with diabetes^(20,22)^. At the state level, deaths were concentrated in the northern and central regions of Maranhão^(24)^.

**Critical synthesis:** Overall, the studies consistently indicate an overlap between TB and socially vulnerable territories. However, gaps remain in comparative analyses between municipalities and in the integration of territorial and clinical variables.

### Axis 2 - Sociodemographic vulnerabilities

TB occurred more frequently among men of working age with low educational attainment and alcohol consumption, factors associated with re-entry into treatment and treatment abandonment^(16)^. TB/HIV coinfection showed a higher prevalence among men with low educational attainment and was associated with an increased risk of abandonment and death^(17)^. Among older adults, low educational attainment, lack of HIV testing, and the presence of diabetes were prominent^(19)^. Another study identified food insecurity and undernutrition as aggravating factors, particularly affecting individuals identified as mixed race^(26)^.

**Critical synthesis:** The evidence reinforces the impact of social and racial inequalities on the dynamics of the disease. Nevertheless, there is a lack of intersectional analyses that integrate different social and clinical dimensions to explain the persistence of TB.

### Axis 3 - Access to and organization of health services

The structure of health services directly influenced diagnosis and treatment. In Caxias, favorable indicators were observed, such as timely care and guaranteed access to medications^(21)^. In São Luís, hospital-based diagnosis predominated, indicating limitations in primary healthcare as the main point of entry^(25)^. At the state level, municipalities with better-structured PHC Units showed greater capacity for case detection^(18)^.

**Critical synthesis:** The studies highlight that strengthening primary healthcare is crucial for addressing TB. Weaknesses persist in integration between levels of care, compromising continuity of care.

### Axis 4 - Conditions and social protection in treatment

Socioeconomic inequalities were associated with a higher risk of death from TB in municipalities in Maranhão^(24)^. Conversely, in São Luís, social benefits were associated with clinical improvement and greater treatment adherence^(26)^.

**Critical synthesis:** Social protection played a fundamental role in therapeutic outcomes and adherence. However, there remains a scarcity of evidence on the reach of different social policies and their effects on the care trajectories of people with TB.

## DISCUSSION

This mixed-methods systematic review sought to synthesize evidence on social, economic, territorial, and healthcare access factors related to the occurrence and outcomes of TB in Maranhão, a Brazilian state with high endemicity. The results indicated that the disease is not homogeneously distributed, concentrating in territories marked by greater social and structural vulnerability. Among the main determinants identified were precarious housing conditions, low educational attainment, food insecurity, comorbidities, racial inequalities, and limitations in the problem-solving capacity of Primary Health Care (PHC).

From a territorial perspective, the high concentration of cases and deaths in São Luís (15) and Imperatriz^(20,22)^ highlights the overlap between urban inequalities and structural weaknesses, a pattern already described in large Latin American urban centers^(24)^. A similar situation has been reported in Ceará, where clusters of high TB incidence were associated with precarious urbanization and social vulnerability, confirming the relationship between territorial inequality and a higher disease burden^(27)^.

At the national level, studies indicate that the Northeast presents average TB mortality rates higher than those in the South and Central-West, reflecting regional disparities in infrastructure and access to healthcare services. Between 2005 and 2019, mortality in the Northeast was approximately 2.9 per 100,000 inhabitants, compared with 1.4 per 100,000 in the South^(28)^. The literature suggests that while regions with greater PHC coverage and better social conditions show stability in indicators, contexts such as the Northeast require continuous and integrated investments to reduce negative outcomes^(29)^.

In the sociodemographic domain, men of working age, individuals with low educational attainment, and those self-identified as mixed race or Black showed a higher likelihood of treatment abandonment or unfavorable outcomes, a pattern well established in national investigations^(30-32)^. The predominance of these groups in the analyzed studies suggests that TB remains a marker of social and racial inequalities, requiring targeted care strategies and social protection.

Regarding the organization of services, findings from Caxias and São Luís revealed a relevant contrast: while municipalities with well-structured PHC Units demonstrated greater capacity for early detection^(18,21)^, the predominance of hospital-based diagnoses in the capital indicates weaknesses in PHC as the main entry point to the health system^(25)^. This dynamic has also been reported in national studies, which identify PHC as a strategic axis for TB control, provided it is articulated with active case finding and continuous follow-up^(33-35)^. Excessive reliance on hospitalization as a diagnostic pathway reinforces delays in detection, increasing community transmission and severe outcomes.

Another critical point concerns social inequalities reflected in higher TB mortality rates in the most vulnerable municipalities of Maranhão^(24)^. This pattern is repeated at the national level, especially in the North and Northeast regions, in contrast to the South and Southeast, where better structural conditions favor greater therapeutic adherence and more positive outcomes^(36,37)^. This heterogeneity underscores that addressing TB requires measures beyond the health sector, integrating social protection, urban policies, and poverty reduction strategies.

In this context, the persistence of TB in Maranhão can be understood as the product of the interaction between limitations in PHC and the fragility of local public policies. By bringing together dispersed studies, this review broadens understanding of the disease in a highly vulnerable territory and provides support for managers and health professionals. Beyond reinforcing the infectious nature of TB, the findings point to the need for intersectoral actions that consider social determinants, with nursing playing a central role in PHC, both in early detection and in adherence to supervised treatment.

### Study limitations

This study has limitations that should be considered. The inclusion of only one case-control study and, in particular, the absence of prospective cohort investigations limit more robust causal inferences. Qualitative production remains incipient, which restricts the understanding of subjective experiences related to illness and TB treatment. The heterogeneity of methods and indicators precluded the performance of a meta-analysis, restricting the synthesis to an interpretive qualitative approach. In addition, the geographic concentration of studies in municipalities such as São Luís, Imperatriz, and Caxias may limit generalizability to other regions of the state. Potential selection biases should also be considered due to the language restriction (Portuguese, English, and Spanish) and the time frame (2014-2025), as well as publication bias, since only indexed articles were included.

### Contributions to nursing, health, and public policy

This systematic review contributes to public health and nursing by demonstrating how social, territorial, and structural factors influence the occurrence and outcomes of TB in contexts of high vulnerability. For nursing practice, the results support strategies in PHC aimed at early detection, strengthening the therapeutic relationship, and adherence to supervised treatment. For public health, the findings reinforce the need for integration between epidemiological surveillance, active case finding, and intersectoral interventions to reduce social and health disparities. In the field of public policy, the results point to the urgency of prioritizing more vulnerable territories, expanding PHC coverage, monitoring social indicators, and integrating social protection policies into TB care. In this way, the study provides actionable evidence for managers and professionals in formulating more effective and context-sensitive responses.

## CONCLUSIONS

The results of this review study show that social, economic, and territorial factors exert a decisive influence on the occurrence and outcomes of TB in Maranhão, concentrating the disease in vulnerable urban territories and among historically neglected populations. The analysis indicates that weaknesses in PHC and structural inequalities hinder early diagnosis and compromise treatment adherence, while social protection policies contribute positively to continuity of care.

These findings reinforce the need for intersectoral responses that integrate health, social assistance, and territorial planning, with priority given to higher-risk areas. It is recommended to expand PHC coverage and problem-solving capacity, strengthen surveillance actions, and incorporate social indicators into TB monitoring. For nursing, the strategic role in active case finding, direct treatment follow-up, and articulation with social support networks is emphasized, all of which are essential for reducing inequities and effectively addressing the disease.

## Data Availability

Not applicable.
