# Supplementary material for: Tuberculosis and social aspects in a Brazilian state of high vulnerability: a systematic review
Source: Rev Bras Enferm. 2026 Aug 21;79:e20250391. doi: 10.1590/0034-7167-2025-0391 (PMC13497353; doi:10.1590/0034-7167-2025-0391)
Supplement: Translation

# Translation: Tuberculose e aspectos sociais em um estado brasileiro de alta vulnerabilidade: revisão sistemática

**DOI:** 10.1590/0034-7167-2025-0391pt

**Authors:** Elivelton Sousa Montelo, Amanda Silva de Oliveira, Zulimar Márita Ribeiro Rodrigues, Ana Hélia de Lima Sardinha

**Affiliations:** I Universidade Federal do Maranhão. São Luís, Maranhão, Brasil

**Keywords:** Tuberculose, Iniquidade em Saúde, Determinantes Sociais da Saúde, Acesso aos Serviços de Saúde, Revisão Sistemática

## Abstract

**Objetivos:**

sintetizar evidências sobre fatores sociais, econômicos, territoriais e de acesso à saúde associados à ocorrência e aos desfechos da tuberculose no Maranhão.

**Métodos:**

revisão sistemática de métodos mistos, registrada no PROSPERO e conduzida conforme o PRISMA-2020. Realizou-se a busca em maio de 2025, com estratégia SPIDER. Foram incluídos estudos originais publicados entre 2014 e 2025, em português, inglês ou espanhol; excluíram-se duplicados e trabalhos sem foco na população-alvo. A qualidade metodológica e o risco de viés foram avaliados pelo Mixed

**Methods:**

Appraisal Tool.

**Resultados:**

dos 94 estudos identificados, 12 foram incluídos. Determinaram-se quatro eixos temáticos: distribuição espacial e áreas de risco; vulnerabilidades sociodemográficas; organização e acesso aos serviços; e condições e proteção social no tratamento. As desigualdades estruturais associaram-se a maior incidência, abandono do tratamento e barreiras no diagnóstico.

**Conclusões:**

a persistência da tuberculose no Maranhão relaciona-se a vulnerabilidades sociais e territoriais, exigindo políticas intersetoriais, fortalecimento da atenção primária e monitoramento de indicadores sociais.

## INTRODUÇÃO

**Quadro 1:** Estratégias de busca utilizadas nas bases de dados, São Luís, Maranhão, Brasil, 2025

Base de dados	Estratégia de busca aplicada	Campo/filtro aplicado
PubMed/MEDLINE	Tuberculosis[Title/Abstract] AND Maranhão[Title/Abstract]	Título/Resumo; sem filtro de estudo
Scopus	TITLE-ABS(Tuberculosis^*^) AND TITLE-ABS(Maranhão)	Título/Resumo; sem filtro
SciELO	Tuberculose AND Maranhão	Campo de busca padrão; idioma: pt/es/em
PsycINFO	TI(Tuberculosis^*^) AND TI(Maranhão)	Título/Resumo; sem filtro
Embase	‘tuberculosis’:ab,ti AND ‘Maranhao’:ab,ti	Título/Resumo; filtro: humanos
BVS (Biblioteca Virtual em Saúde)	tw:(Tuberculose^*^) AND tw:(Maranhão)	Título/Resumo (tw); filtro: humanos; 2014-2025

Nota: Quando aplicável, foi utilizada truncagem (^*^) para ampliar a recuperação de termos relacionados, de acordo com a sintaxe de cada base.

A tuberculose (TB) é uma doença infecciosa causada pelo Mycobacterium tuberculosis, que acomete prioritariamente os pulmões, embora possa comprometer outros órgãos e sistemas. A transmissão ocorre por via aérea, a partir da exposição a aerossóis eliminados por indivíduos com TB pulmonar ativa^(1)^. Em 2022, a TB permaneceu entre as principais causas de morte por doenças infecciosas no mundo, com 10,6 milhões de novos casos e 1,3 milhão de óbitos, segundo a Organização Mundial da Saúde^(2)^.

No Brasil, a TB mantém forte impacto na saúde pública. Em 2023, foram registrados mais de 80 mil novos casos, resultando em um coeficiente de incidência de 37,0 por 100 mil habitantes, além de 5.845 óbitos^(3)^. Apesar da ampliação do diagnóstico e do tratamento, a doença persiste concentrada em territórios marcados por desigualdades sociais e estruturais, como pobreza, moradia inadequada e barreiras de acesso aos serviços de saúde^(4)^.

No contexto do Maranhão, essas condições são ainda mais acentuadas. O estado apresenta elevados índices de pobreza, baixa escolaridade, precariedade habitacional e acesso limitado a saneamento e à atenção primária, fatores que favorecem a transmissão e dificultam o controle da TB^(5)^. Em 2023, foram notificados 2.808 casos, levando o Ministério da Saúde a incluí-lo entre os estados prioritários para ações de enfrentamento^(6)^.

Embora a literatura aponte associações entre determinantes sociais e a TB, os estudos disponíveis no Maranhão encontram-se dispersos, heterogêneos e, em geral, com foco em aspectos isolados^(7,8)^, por vezes epidemiológicos, apenas socioeconômicos ou relacionados à organização dos serviços. Não se encontraram sínteses integradas que permitam compreender de forma abrangente como fatores sociais, econômicos, territoriais e de acesso à saúde influenciam simultaneamente os desfechos da TB nesse estado. Diante dessas lacunas científicas, acabam-se gerando limitações à capacidade de gestores e profissionais formularem políticas públicas baseadas em evidências locais, capazes de enfrentar os contextos de vulnerabilidades em torno da TB.

## OBJETIVOS

Sintetizar evidências disponíveis sobre fatores sociais, econômicos, territoriais e de acesso à saúde associados à ocorrência e aos desfechos da TB em populações do Maranhão.

## MÉTODOS

### Desenho e protocolo do estudo

Trata-se de uma revisão sistemática de métodos mistos, registrada no *International Prospective Register of Systematic Reviews* (PROSPERO)^(9)^ sob o número CRD42020214295, e conduzida conforme as diretrizes do P*referred Reporting Items for Systematic Reviews and Meta-Analyses* (PRISMA), 2020^(10)^.

### Estratégia SPIDER e questão norteadora

A pergunta de pesquisa foi estruturada com base na estratégia SPIDER^(11)^, adequada para revisões com dados qualitativos e de métodos mistos:

- S (*Sample*): Populações acometidas pela tuberculose no estado do Maranhão;
- PI (*Phenomenon of Interest*): Fatores sociais, econômicos, territoriais, epidemiológicos e de acesso à saúde;
- D (*Design*): Estudos qualitativos, quantitativos e de métodos mistos;
- E (*Evaluation*): Percepções, experiências, associações ou efeitos atribuídos aos fatores estudados;
- R (*Research type*): Publicações em português, inglês ou espanhol.

A busca foi realizada em maio de 2025, nas bases PubMed/MEDLINE, Scopus, SciELO, PsycINFO, Embase e Biblioteca Virtual em Saúde (BVS). De acordo com a estratégia SPIDER, formulou-se a seguinte questão norteadora: Quais fatores sociais, econômicos, territoriais e de acesso à saúde estão associados à ocorrência e aos desfechos da tuberculose, como incidência, mortalidade, adesão ao tratamento, abandono e diagnóstico oportuno, no Maranhão, estado brasileiro de alta endemicidade, segundo evidências publicadas entre 2014 e 2025?

Estratégias preliminares mais amplas foram testadas, mas a busca final priorizou termos aplicados ao título e ao resumo, como *Tuberculosis* AND Maranhão, pela maior sensibilidade. A lógica foi adaptada às demais bases segundo a sintaxe de cada uma. A busca foi realizada utilizando a estratégia SPIDER, com descritores controlados e não controlados combinados por operadores booleanos (AND, OR) e truncagem (*). Os termos foram aplicados ao título e ao resumo, opção adotada para aumentar a especificidade e evitar a recuperação excessiva de estudos irrelevantes, considerando a ampla dispersão conceitual relacionada aos determinantes sociais da saúde.

### Critérios de inclusão e exclusão

Foram incluídos estudos primários sobre TB, com delineamento qualitativo, quantitativo ou de métodos mistos, desenvolvidos exclusivamente no estado do Maranhão, publicados entre janeiro de 2014 e maio de 2025, em português, inglês ou espanhol, que abordassem fatores sociais, econômicos, territoriais ou de acesso à saúde relacionados à doença em seres humanos. O recorte temporal de 2014 a 2025 foi adotado por contemplar a última década de publicações e permitir a análise de evidências recentes, inclusive aquelas produzidas após as atualizações mais recentes do Plano Nacional de Controle da Tuberculose (PNCT)^(3)^.

Foram excluídos estudos duplicados, revisões, ensaios teóricos, editoriais, teses e dissertações não publicadas; trabalhos com foco estritamente clínico, laboratorial ou biomolecular, sem interface com fatores sociais ou contextuais; pesquisas voltadas exclusivamente à proposição ou validação de instrumentos; estudos envolvendo populações muito específicas, como portadores de comorbidades raras, cuja análise dificultasse a interpretação dos determinantes sociais em sentido mais amplo; e artigos que não atenderam a critérios mínimos de qualidade metodológica, avaliados pelo *Mixed Methods Appraisal Tool* (MMAT) (versão 2018).

### Seleção dos estudos

A seleção dos estudos foi conduzida em três etapas sucessivas: leitura de títulos, resumos e textos completos. O processo foi realizado de forma independente por duas revisoras (E.S.M. e A.H.L.S.), com resolução de divergências por uma terceira pesquisadora (Z.M.R.R.). Os registros duplicados foram previamente identificados e removidos com o auxílio do *software* Mendeley^©^, garantindo maior precisão na triagem. O processo de identificação, triagem, elegibilidade e inclusão final está detalhado no fluxograma elaborado conforme as diretrizes PRISMA 2020.

### Avaliação da qualidade metodológica

A qualidade metodológica e o risco de viés dos estudos foram avaliados com o MMAT, versão 2018^(12)^. Essa ferramenta utiliza critérios específicos conforme o delineamento, considerando aspectos como clareza dos objetivos, adequação metodológica, representatividade da amostra, validade das medidas e consistência entre resultados e conclusões.

Os pesquisadores responsáveis passaram previamente por treinamento, utilizando planilha padronizada no *Microsoft Excel* ^®^, de modo a assegurar uniformidade na aplicação dos critérios. A avaliação foi conduzida de forma independente por dois revisores, com discussão posterior para consenso em casos de discordância. A escolha pelo MMAT justifica-se por sua abrangência e aplicabilidade em revisões sistemáticas que integram diferentes abordagens metodológicas.

### Extração e análise dos dados

Os dados foram extraídos para uma planilha padronizada no *Microsoft Excel* ^®^, contendo autores, ano de publicação, local do estudo, delineamento, abordagem metodológica, população, variáveis investigadas, principais achados e avaliação pelo MMAT. A extração foi realizada de forma cega por dois revisores, que receberam treinamento prévio no uso da planilha, assegurando uniformidade no processo e resolução de divergências por consenso, com a participação de uma terceira pesquisadora quando necessário.

A análise seguiu o método de Braun & Clarke^(13,14)^, estruturado em seis etapas: (1) familiarização com os resultados dos estudos incluídos; (2) geração de códigos iniciais; (3) busca por temas; (4) revisão dos temas; (5) definição e nomeação; e (6) síntese dos achados em relatório. Para os estudos quantitativos, foi realizada síntese descritiva dos principais indicadores (incidência, mortalidade, adesão, abandono e diagnóstico oportuno), apresentados em quadro/tabulação. Já os estudos qualitativos e de métodos mistos foram integrados por meio de análise interpretativa. Devido à heterogeneidade de delineamentos e desfechos, não foi possível realizar metanálise.

## RESULTADOS

Foram identificados 94 estudos nas bases de dados selecionadas. Após a remoção de 68 duplicatas, 26 artigos foram submetidos à triagem por leitura dos títulos e resumos. Destes, 14 foram excluídos por não atenderem aos critérios de elegibilidade, resultando em 12 estudos incluídos na revisão. O processo de seleção está detalhado no fluxograma PRISMA (Figura 1).

**Figura 1:** Fluxograma de seleção dos estudos, elaborado a partir da recomendação *Preferred Reporting Items for Systematic Reviews and Meta-Analyses*, São Luís, Maranhão, Brasil, 2025

Dos 12 estudos incluídos na presente revisão, 11 apresentavam delineamento quantitativo, sendo cinco do tipo ecológico, dos quais quatro utilizaram análise espacial, quatro transversais e um estudo caso-controle. Além disso, foi identificado um estudo com abordagem de métodos mistos. Mesmo realizando as buscas a partir do ano de 2014, os estudos elegíveis que compõem a inclusão final foram publicados entre 2015 e 2025. Quanto ao idioma de publicação, dois artigos estavam disponíveis apenas em português, seis em inglês e quatro em ambas as línguas, e nenhum em espanhol. A qualidade metodológica foi avaliada com base nos escores do MMAT, sendo que seis estudos obtiveram 100%, cinco alcançaram 80% e um apresentou 60%. As principais características metodológicas e temáticas dos estudos incluídos estão sistematizadas no Quadro 2, contemplando informações sobre autoria, território de realização, delineamento e tamanho da amostra, variáveis investigadas, principais resultados e qualidade metodológica avaliada pelo MMAT.

**Quadro 2:** Características metodológicas e principais resultados dos estudos incluídos na revisão

Autoria (ano)	Território	Delineamento / nº de participantes	Principais variáveis	Principais resultados	Qualidade (MMAT)
Santos Neto et al. (2015)^(15)^	São Luís-MA	Ecológico com análise espacial; n=221 óbitos (2008-2012)	Aglomerados espaciais e espaço-temporais de óbitos por TB	Clusters significativos: 5,8/100.000 hab./ano (RR=3,87) e 0,4/100.000 hab./ano (RR=0,10). Cluster espaço-temporal: nov/2008-abr/2011 (8,1/100.000 hab./ano; RR=3,0).	80%
Silva et al. (2015)^(16)^	Municípios prioritários do MA (8)	Transversal; n=2.277 casos (2005-2010)	Fatores associados a retratamento por recidiva e reingresso	Homens, 20-39 anos, <8 anos de estudo e alcoolismo associados a reingresso; dependentes de álcool com maior chance de novo abandono (OR=5,96; p<0,001).	100%
Pereira et al. (2018)^(17)^	Maranhão	Transversal; n=4.553 casos (2001-2011)	Fatores associados à coinfecção TB/HIV	Prevalência de coinfecção TB/HIV: 15,1%. Sexo masculino, 29-30 anos, baixa escolaridade e abandono/óbito associados.	100%
Costa et al. (2018)^(18)^	Municípios prioritários e macrorregiões do MA	Ecológico; n=1.706 casos novos de TB pulmonar e 1.365 UBSs (2012)	Características das UBS e detecção de casos de TB	Maior adequação das UBS associou-se a maior detecção de casos (IDR=1,61; IC95%: 1,01-2,60).	100%
Silva et al. (2019)^(19)^	Maranhão	Transversal; n=1.505 idosos ≥60 anos (2010-2015)	Perfil sociodemográfico e clínico de idosos com TB	Escolaridade <8 anos, alta sem cura, ausência de teste HIV e diabetes associados à TB em idosos.	100%
Andrade et al. (2020)^(20)^	Imperatriz-MA	Ecológico com análise espacial; n=413 casos (2013-2018)	Distribuição espacial e desfechos no tratamento da TB	Maiores densidades de cura, óbito e abandono na região central do município.	80%
Müller et al. (2021)^(21)^	Caxias-MA	Transversal; n=100 pacientes de 10 UBSs (2018)	Acesso ao tratamento de TB na atenção primária à saúde	Indicadores de acesso: tratamento na unidade mais próxima (100%), consulta ≤24h (91%), acesso a medicamentos (99%).	60%
Sousa et al. (2021)^(22)^	Imperatriz-MA	Ecológico com análise espacial; n=721 casos (2009-2018)	Vulnerabilidade territorial à comorbidade TB-DM	Três clusters de alto risco (p<0,05) com RR=4,00; 5,10; 6,10 em 11 setores urbanos.	80%
Aragão et al. (2023)^(23)^	São Luís-MA	Transversal; n=7.948 casos (2010-2020)	Organização do sistema de saúde para diagnóstico de TB	1.608 casos na APS e 6.340 em hospitais; diferenças significativas entre exames (p<0,05).	80%
Santos et al. (2023)^(24)^	Maranhão	Ecológico com análise espacial; n=949 óbitos (2010-2015)	Distribuição espacial e indicadores socioeconômicos relacionados a óbitos por TB	≈12% dos municípios com >75% de chance de óbito; concentração no Norte e centro do estado.	80%
Aragão et al. (2024)^(25)^	São Luís-MA	Método misto sequencial explanatório; n=7.958 casos (2010-2019)	Áreas de risco e proteção social no tratamento da TB	Treze clusters de risco identificados; benefícios sociais associados à melhora.	100%
Mendes et al. (2025)^(26)^	São Luís-MA	Caso-controle; n=138 indivíduos (65 TB, 73 controles)	Fatores nutricionais e insegurança alimentar	TB associada ao baixo peso (26,2%), insegurança alimentar (64%) e baixa escolaridade.	100%

*APS - Atenção Primária à Saúde; DM - Diabetes mellitus; HIV - Vírus da Imunodeficiência Humana; IC - Intervalo de confiança; IDR - Razão de densidade de incidência; IMC - Índice de Massa Corpórea; MA - Maranhão; OR - Odds ratios; RR - Risco relativo; TB - Tuberculose; TB-DM - Tuberculose e diabetes mellitus; TB/HIV - Tuberculose e Vírus da Imunodeficiência Humana; UBSs - Unidades Básicas de Saúde.*

Com base na análise temática de Braun & Clarke^(13,14)^ e considerando o componente *Phenomenon of Interest* (PI) da estratégia SPIDER^(11)^, centrado em fatores sociais, econômicos, territoriais, epidemiológicos e de acesso à saúde, os resultados foram organizados em quatro eixos. O processo envolveu codificação, agrupamento e revisão cíclica dos dados, permitindo identificar padrões recorrentes e sintetizar determinantes que influenciam a ocorrência, o diagnóstico e o tratamento da TB no Maranhão. Os eixos identificados foram: (1) Distribuição espacial e áreas de risco; (2) Vulnerabilidades sociodemográficas; (3) Acesso e organização dos serviços de saúde; e (4) Condições e proteção social no tratamento.

### Eixo 1 - Distribuição espacial e áreas de risco

Estudos espaciais mostraram concentração desigual de casos e óbitos por TB. Em São Luís, observaram-se clusters de mortalidade em períodos e regiões específicas^(15)^. Na cidade de Imperatriz, houve maior densidade de cura, abandono e óbito em áreas centrais vulneráveis, além de clusters de risco para TB associada ao diabetes^(20,22)^. Em escala estadual, óbitos concentraram-se no Norte e no centro do Maranhão^(24)^.

**Síntese crítica:** De forma consistente, os trabalhos indicam sobreposição da TB a territórios socialmente vulneráveis. Persistem, no entanto, lacunas em análises comparativas entre municípios e na integração de variáveis territoriais e clínicas.

### Eixo 2 - Vulnerabilidades sociodemográficas

A TB ocorreu com maior frequência entre homens em idade produtiva, com baixa escolaridade e consumo de álcool, fatores associados ao reingresso e ao abandono^(16)^. A coinfecção TB/HIV mostrou maior prevalência em homens com baixa escolaridade, associada a risco elevado de abandono e óbito^(17)^. Em idosos, destacaram-se baixa escolaridade, ausência de testagem para HIV e presença de diabetes^(19)^. Outro estudo apontou insegurança alimentar e desnutrição como agravantes, afetando especialmente pessoas pardas^(26)^.

**Síntese crítica:** As evidências encontradas reforçam o impacto das desigualdades sociais e raciais na dinâmica da doença. Ainda assim, faltam análises interseccionais que articulem diferentes dimensões sociais e clínicas para explicar a persistência da TB

### Eixo 3 - Acesso e organização dos serviços de saúde

A estrutura dos serviços influenciou diretamente o diagnóstico e tratamento. Em Caxias, verificaram-se bons indicadores, como atendimento rápido e acesso garantido a medicamentos^(21)^. Em São Luís, predominou o diagnóstico hospitalar, apontando limitações na atenção primária como porta de entrada^(25)^. Em nível estadual, municípios com UBSs mais bem estruturadas apresentaram maior capacidade de detecção de casos^(18)^.

**Síntese crítica:** Os estudos destacam que a qualificação da atenção primária é determinante para o enfrentamento da TB. Permanecem fragilidades na integração entre níveis assistenciais, o que compromete a continuidade do cuidado.

### Eixo 4 - Condições e proteção social no tratamento

As desigualdades socioeconômicas estiveram associadas a maior risco de óbito por TB em municípios maranhenses^(24)^. Em contrapartida, em São Luís, benefícios sociais foram relacionados à melhora clínica e maior adesão ao tratamento^(26)^.

**Síntese crítica:** A proteção social demonstrou papel fundamental na evolução terapêutica e na adesão. Entretanto, ainda há escassez de evidências sobre o alcance de diferentes políticas sociais e seus efeitos sobre a trajetória das pessoas com TB.

## DISCUSSÃO

Esta revisão sistemática de métodos mistos buscou sintetizar evidências sobre fatores sociais, econômicos, territoriais e de acesso à saúde relacionados à ocorrência e aos desfechos da TB no Maranhão, estado brasileiro de alta endemicidade. Os resultados indicaram que a doença não se distribui de forma homogênea, concentrando-se em territórios marcados por maior vulnerabilidade social e estrutural. Entre os principais determinantes, destacaram-se precariedades habitacionais, baixa escolaridade, insegurança alimentar, comorbidades, desigualdades raciais e limitações na resolutividade da Atenção Primária à Saúde (APS).

No campo territorial, a elevada concentração de casos e óbitos em São Luís^(15)^ e Imperatriz^(20,22)^ evidencia a sobreposição de desigualdades urbanas e fragilidades estruturais, padrão já descrito em grandes centros urbanos latino-americanos^(24)^. Situação semelhante foi relatada no Ceará, onde clusters de alta incidência de TB foram associados à urbanização precária e à vulnerabilidade social, confirmando a relação entre desigualdade territorial e maior carga da doença^(27)^.

Em perspectiva nacional, estudos apontam que o Nordeste apresenta taxas médias de mortalidade por TB superiores às do Sul e Centro-Oeste, reflexo das disparidades regionais de infraestrutura e acesso aos serviços de saúde. Entre 2005 e 2019, a mortalidade no Nordeste foi de cerca de 2,9/100 mil habitantes, contra 1,4/100 mil no Sul^(28)^. A literatura indica que, enquanto regiões com maior cobertura da APS e melhores condições sociais apresentam estabilidade nos indicadores, contextos como o Nordeste exigem investimentos contínuos e integrados para reduzir desfechos negativos^(29)^.

No eixo sociodemográfico, observou-se que homens em idade produtiva, pessoas com baixa escolaridade e indivíduos autodeclarados pardos ou pretos apresentaram maior probabilidade de abandono ou desfechos desfavoráveis, padrão já consolidado em investigações nacionais^(30-32)^. O predomínio desses grupos nos estudos analisados sugere que a TB se mantém como marcador de desigualdades sociais e raciais, exigindo estratégias direcionadas de cuidado e proteção social.

Em relação à organização dos serviços, os achados de Caxias e São Luís mostraram contraste relevante: enquanto municípios com Unidades Básicas de Saúde (UBSs) estruturadas demonstraram maior capacidade de detecção precoce^(18,21)^, a predominância de diagnósticos hospitalares na capital aponta fragilidades da APS como porta de entrada do sistema^(25)^. Essa dinâmica também foi relatada em estudos nacionais, que indicam a APS como eixo estratégico no controle da TB, desde que articulada à busca ativa e ao acompanhamento contínuo^(33-35)^. A dependência excessiva da hospitalização como meio diagnóstico reforça atrasos na detecção, ampliando a transmissão comunitária e os desfechos graves.

Outro ponto crítico refere-se às desigualdades sociais que se refletem em maiores taxas de mortalidade nos municípios mais vulneráveis do Maranhão^(24)^. Esse padrão repete-se em escala nacional, especialmente nas regiões Norte e Nordeste, em contraste com o Sul e Sudeste, onde melhores condições estruturais favorecem maior adesão terapêutica e resultados mais positivos^(36,37)^. Essa heterogeneidade evidencia que o enfrentamento da TB requer medidas além do setor saúde, articulando proteção social, políticas urbanas e estratégias de redução da pobreza.

Diante disso, a persistência da TB no Maranhão pode ser entendida como produto da interação entre limitações na APS e fragilidade das políticas públicas locais. Ao reunir estudos dispersos, esta revisão amplia a compreensão sobre a doença em um território de alta vulnerabilidade e fornece subsídios para gestores e profissionais. Mais do que reforçar o caráter infeccioso da TB, os resultados apontam a necessidade de ações intersetoriais que considerem determinantes sociais, com papel central da enfermagem na APS, tanto na detecção precoce quanto na adesão ao tratamento supervisionado.

### Limitações do estudo

Este trabalho apresenta limitações que devem ser consideradas. A presença de apenas um estudo de caso-controle e, principalmente, a ausência de investigações de coortes prospectivas restringem inferências causais mais robustas. A produção qualitativa ainda é incipiente, o que limita a compreensão de experiências subjetivas relacionadas ao adoecimento e ao tratamento da TB. A heterogeneidade de métodos e indicadores impossibilitou a realização de metanálise, restringindo a síntese a uma abordagem qualitativa interpretativa. Ademais, a concentração geográfica dos estudos em municípios como São Luís, Imperatriz e Caxias pode limitar a generalização para outras regiões do estado. Deve-se ainda considerar possíveis vieses de seleção, pelo recorte de idiomas (português, inglês e espanhol) e do período (2014-2025), bem como o viés de publicação, já que apenas artigos indexados foram incluídos.

### Contribuições para as áreas da enfermagem, saúde e políticas públicas

Esta revisão sistemática contribui para a saúde coletiva e a enfermagem ao evidenciar como fatores sociais, territoriais e estruturais influenciam a ocorrência e os desfechos da TB em contextos de alta vulnerabilidade. Para a prática da enfermagem, os resultados subsidiam estratégias na APS voltadas à detecção precoce, ao fortalecimento do vínculo terapêutico e à adesão ao tratamento supervisionado. Para a saúde coletiva, reforçam a necessidade de integração entre vigilância epidemiológica, busca ativa e intervenções intersetoriais que reduzam as disparidades sociais e em saúde. No campo das políticas públicas, apontam para a urgência de priorizar territórios mais vulneráveis, ampliar a cobertura da APS, monitorar indicadores sociais e integrar políticas de proteção social ao cuidado em TB. Dessa forma, o estudo fornece evidências aplicáveis para gestores e profissionais na formulação de respostas mais efetivas e contextualizadas.

## CONCLUSÕES

Os resultados deste estudo de revisão evidenciam que fatores sociais, econômicos e territoriais exercem influência decisiva sobre a ocorrência e os desfechos da TB no Maranhão, concentrando a doença em territórios urbanos vulneráveis e entre populações historicamente negligenciadas. A análise mostra que a fragilidade da APS e as desigualdades estruturais dificultam o diagnóstico precoce e comprometem a adesão ao tratamento, enquanto políticas de proteção social contribuem positivamente para a continuidade do cuidado.

Essas evidências reforçam a necessidade de respostas intersetoriais que integrem saúde, assistência social e planejamento territorial, com prioridade para áreas de maior risco. Recomenda-se ampliar a cobertura e a resolutividade da APS, fortalecer ações de vigilância e incorporar indicadores sociais no monitoramento da TB. Para a enfermagem, destaca-se o papel estratégico na busca ativa de sintomáticos, no acompanhamento direto do tratamento e na articulação com redes de apoio social, fundamentais para a redução das iniquidades e o enfrentamento efetivo da doença.

## Data Availability

Não se aplica.
